# Architectural and geotechnical aspects affecting earthquake resilience for the antique Egyptian Khufu pyramid

**DOI:** 10.1038/s41598-026-49962-6

**Published:** 2026-05-21

**Authors:** Mohamed ELGabry, Ayman Hamed, Sakuji Yoshimura, Hesham M. Hussein, Mohamed Maklad, Asem Salama

**Affiliations:** 1 Egyptian National Data Center (ENDC), National Research Institute of Astronomy and Geophysics (NRIAG), Helwan, Cairo, 11421 Egypt; 2 Faculty of Petroleum and Mining Engineering, Suez University, Suez, Egypt; 3 Institute of Egyptian Archaeology, Higashi Nippon International University, Iwaki, Japan; 4 General Seismology Laboratory, National Research Institute of Astronomy and Geophysics (NRIAG), Helwan, Cairo, 11421 Egypt; 5 African Disaster Mitigation Research Center (ADMiR), Cairo, Egypt

**Keywords:** Khufu pyramid, Horizontal to the vertical spectral ratio (HVSR), Amplification, Fundamental frequency, Egyptian old kingdom, Engineering, Natural hazards, Solid Earth sciences

## Abstract

The Great Pyramid of Khufu, completed during Egypt’s Old Kingdom (2600–2450 BCE), exhibits the architectural expertise of ancient Pharaonic Egypt. To understand the structural longevity and earthquake resilience of this iconic monument, we carried out a comprehensive ambient noise survey employing horizontal-to-vertical spectral ratio (HVSR) analysis at 37 measurement points distributed throughout the pyramid’s internal chambers, construction blocks, and adjacent soil. Our analysis reveals several critical findings. First, the pyramid exhibits uniform fundamental frequencies (2.0–2.6 Hz) with an average of ~ 2.3 Hz across all structural elements, indicating exceptional homogeneity in dynamic characteristics. Second, this frequency band differs significantly from that of the surrounding soil (~ 0.6 Hz), preventing resonance amplification through soil-structure interaction—a key mechanism protecting the monument during seismic activity. Third, seismic relative amplification increases systematically with elevation up to 48.68 m, but diminishes substantially within the pressure-relieving chambers (48.86–61.07 m), demonstrating how their geometry actively reduces seismic response. Finally, seismic vulnerability assessment of the subsurface foundation yields a low value (kg = 8.2), confirming excellent bearing capacity and minimal earthquake-induced risk. The low seismic vulnerability index estimated for the foundation soils suggests that any future earthquakes are likely to produce only limited damage to the main pyramid body. These findings present compelling quantitative evidence that ancient Egyptian architects possessed profound geotechnical understanding, optimising structure design and site characterisation to assure millennial-scale stability against seismic hazards.

## Introduction

**Fig. 1:**
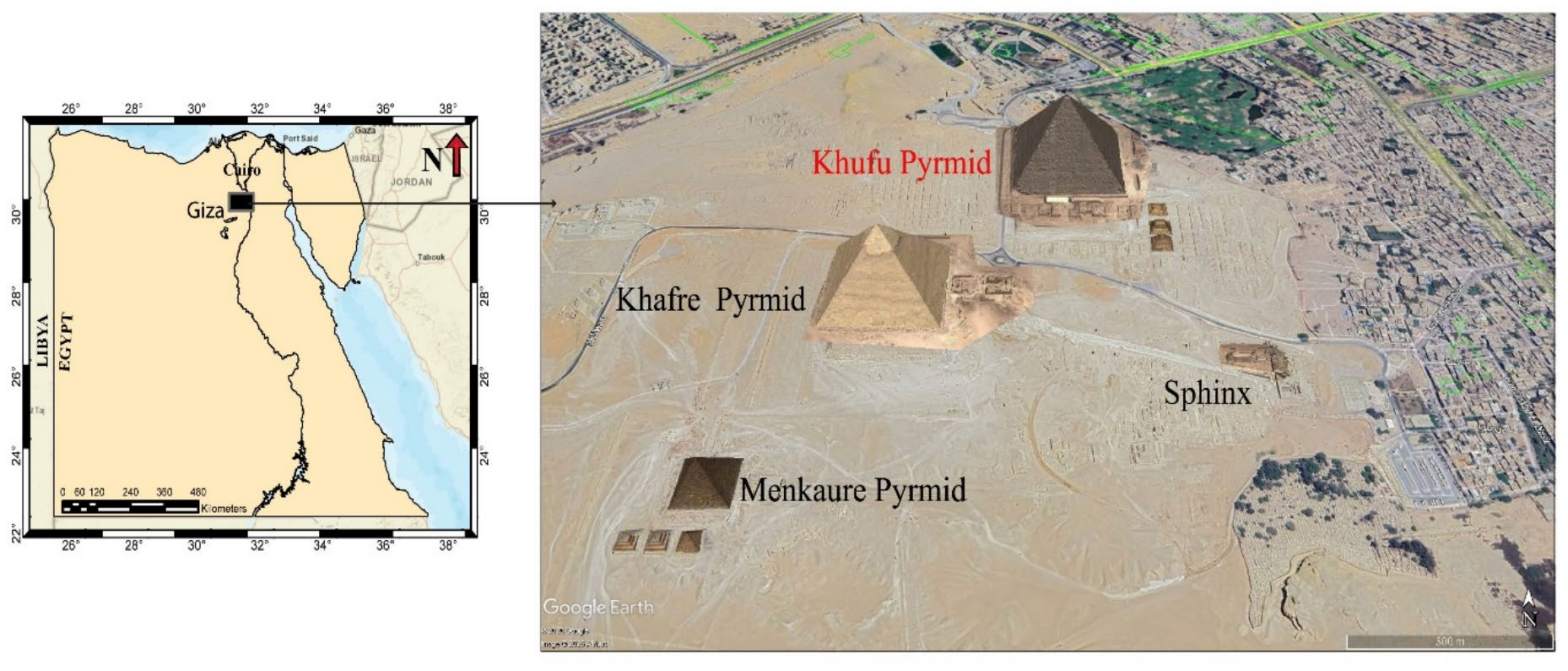
Location map of the Great Khufu Pyramid, Landsat image updated in July 2020 (Google Earth database).

**Fig. 2:**
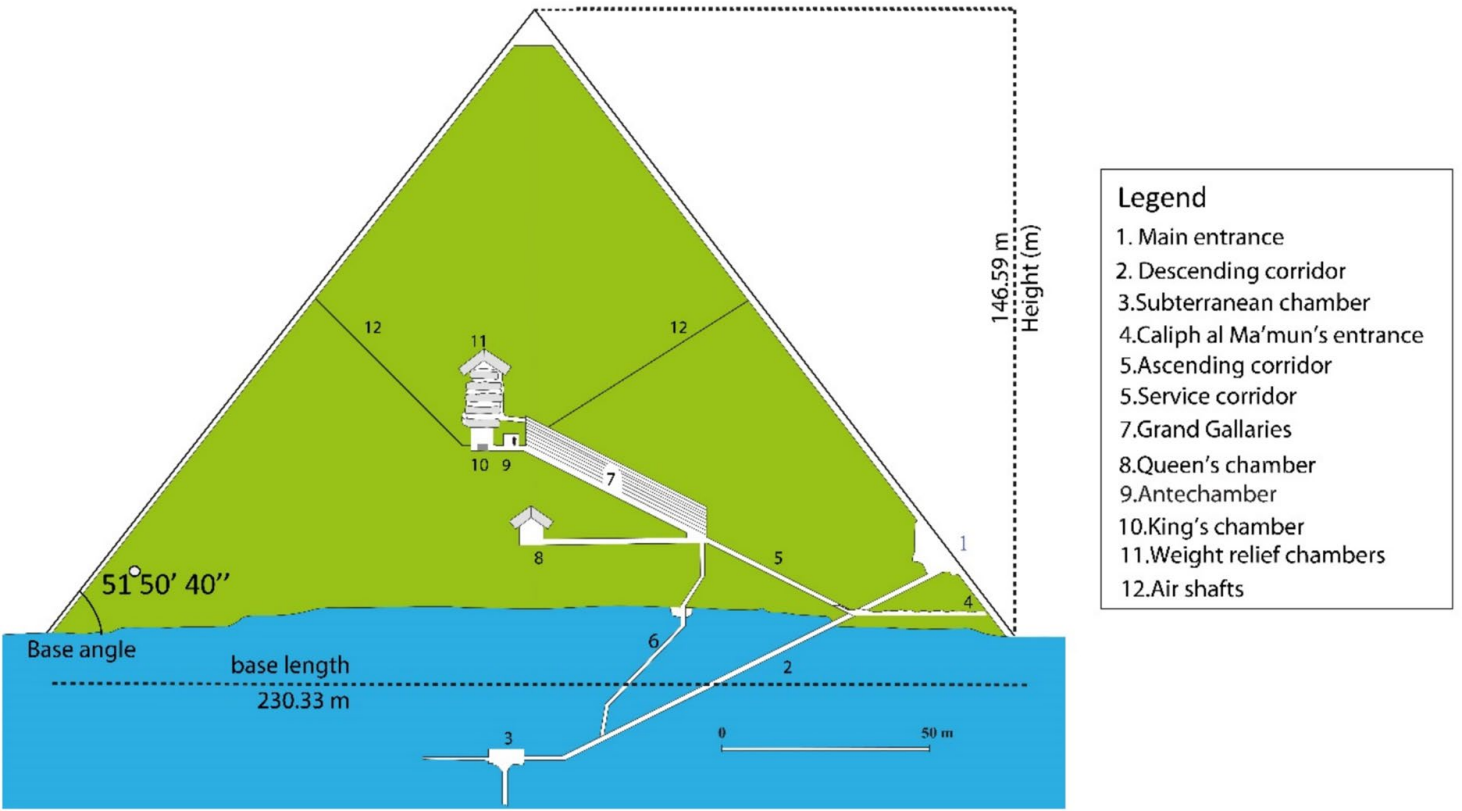
Sketch showing the structure of the Khufu Pyramid.

The Khufu pyramid, or Pyramid of Cheops, was the oldest Pyramid constructed in the northwestern part of the Giza plateau in Cairo during the Old Kingdom (Fig. 1)^1,2^. provided a comprehensive account of the architectural design of the Pyramid of Khufu (Fig. 2). The pyramid was originally constructed to a height of 146.59 m, with a base length of approximately 230.33 m per side and a slope angle of 51° 50′ 40"^1–3^**.** However, current measurements indicate that the present height is about 137 m, reflecting the loss of the original casing stones and apex over time^3,4^. According to^1,2^, the pyramid’s structure consists of a core and a casing of horizontal stones, with supporting blocks interspersed throughout. The Great Pyramid is estimated to contain around 2,300,000 stone blocks, each carefully positioned to achieve its monumental scale and stability^1,2,4^. Internally, Khufu’s pyramid features eight principal elements: the main entrance with descending passage, the entrance created by Caliph al-Ma’mun’s workmen, the Subterranean Chamber, the Grand Gallery, the Queen’s Chamber, the King’s Chamber, the relieving chambers, and the shafts^1–3,5^. This intricate arrangement highlights the advanced engineering and architectural planning characteristic of Old Kingdom pyramid construction^1–4^.

The pyramid area has been affected by numerous earthquakes within an epicentral radius of 80 km, without any serious damage to the main body of the Khufu pyramid over 4600 years. The largest reported earthquake event was on 7 August 1847 with an estimated magnitude of 6.8^6^**.** This event was located near El-Fayoum, ~ 70 km from the Giza pyramids. Later, on 12 October 1992, an earthquake with a magnitude of 5.8 struck Giza again. During this event, several casing stones fell from the top parts of the pyramids^7^.

Desoky and Hendawy^8^ examined the pyramid’s architecture and shape from a civil engineering perspective. They noted that most of the mass is concentrated near the ground and gradually decreases toward the top. The symmetrical design also suggests good balance and centralised mass distribution. They summarized out these features as: (a) Smart design that channels vibration forces safely through the structure; (b) Solid torsion resistance and stiffness to avoid uneven stress; (c) Good damping that calms down shaking and helps prevent resonance; (d) Low height-to-base ratio keeping it stable against tipping; (e) Both centres of mass and resistance are located at, or close to, the same point. This will cause the elimination/ reduction of torsion. (f) Plan dense footprint that resists overturning well; (g) No re-entrant corners to avoid stress buildup; (h) Existence of lateral resisting components along the Perimeter of the structure. This will produce a structural layout with high rigidity and strength.

One of the main parameters which control the dynamic response of a structure is the interaction between natural frequencies inside the structure and the surrounding ground. The dynamic response is defined as the oscillation of the building at a certain vibration frequency as a result of the dynamic motion of an earthquake^9^. This phenomenon is one of the most significant factors governing structural damage during an earthquake. We also examine the relationship between the relative amplification factor of the pyramid’s various structural elements and the different heights of these elements. Ambient noise is simply a natural signal, consisting of an assemblage of body waves and surface waves which exist everywhere^10^**.** These signals may exist either because of human activities, oceanic waves or climatic changes^11^.

The advanced applications of Ambient noise (HVSR) utilised in various studies for multiple purposes are based on Nakamura’s foundational research^12^, including HVSR reliability, ambient vibrations in historical monuments, soil-structure interactions in heritage buildings, and seismic evaluations of masonry structures. Khalil et al.^13^ used the HVSR method to investigate subsurface features for exploring buried tunnel monuments found at 20 m at the southern Saqqara (Zoser pyramid) site.^14^ used the HVSR to evaluate the seismic vulnerability of eight cultural heritage minarets in Cairo, identifying the parameters influencing their seismic behaviour and susceptibility to earthquake damage. Altunışık et al.^15^ examined the seismic behaviour of the Santa Maria Church and its Guesthouse Building located in Trabzon, Turkey, with an emphasis on soil–structure interaction and various earthquake input models. Their findings indicate that the interaction between the soil and structure, along with the types of soil, has considerably influenced the structural responses of both buildings. Coviello et al.^16^ used a smart experimental setup with non-invasive methods like controlled vehicle passes, hammer impacts, and ambient vibration tests—carefully picked to get good signals while protecting the heritage sites. Their results gave accurate, reliable natural frequencies and mode shapes from the big dataset, enabling solid seismic assessments and better prediction of how three-span masonry bridges respond to earthquakes. This helps plan long-term preservation for these valuable, at-risk structures^17^. studied site effects at Egyptian National Seismic Network stations using HVSR inversion combined with the MASW technique. The site-specific amplification data they got helps us better understand local ground conditions and nail down precise site classification and characterisation factors.^18^ used automated HVSR tools for estimating site fundamental frequency and its uncertainty using HVSR curves for IRIS seismic stations. Thabet^19^ studied the specific relationships between bedrock and HVSR resonance frequency using KIK-NET station data from Japan. It emphasises the use of regression analysis and strong validations through extensive data.^20^ used a new global optimisation algorithm called Modified Barnacles Mating Optimiser (MBMO) to improve the accuracy of HVSR and surface wave inversions. It handles full-parametric, transdimensional, and joint inversions better, reducing uncertainty and giving more reliable pictures of subsurface structures.

**Fig. 3:**
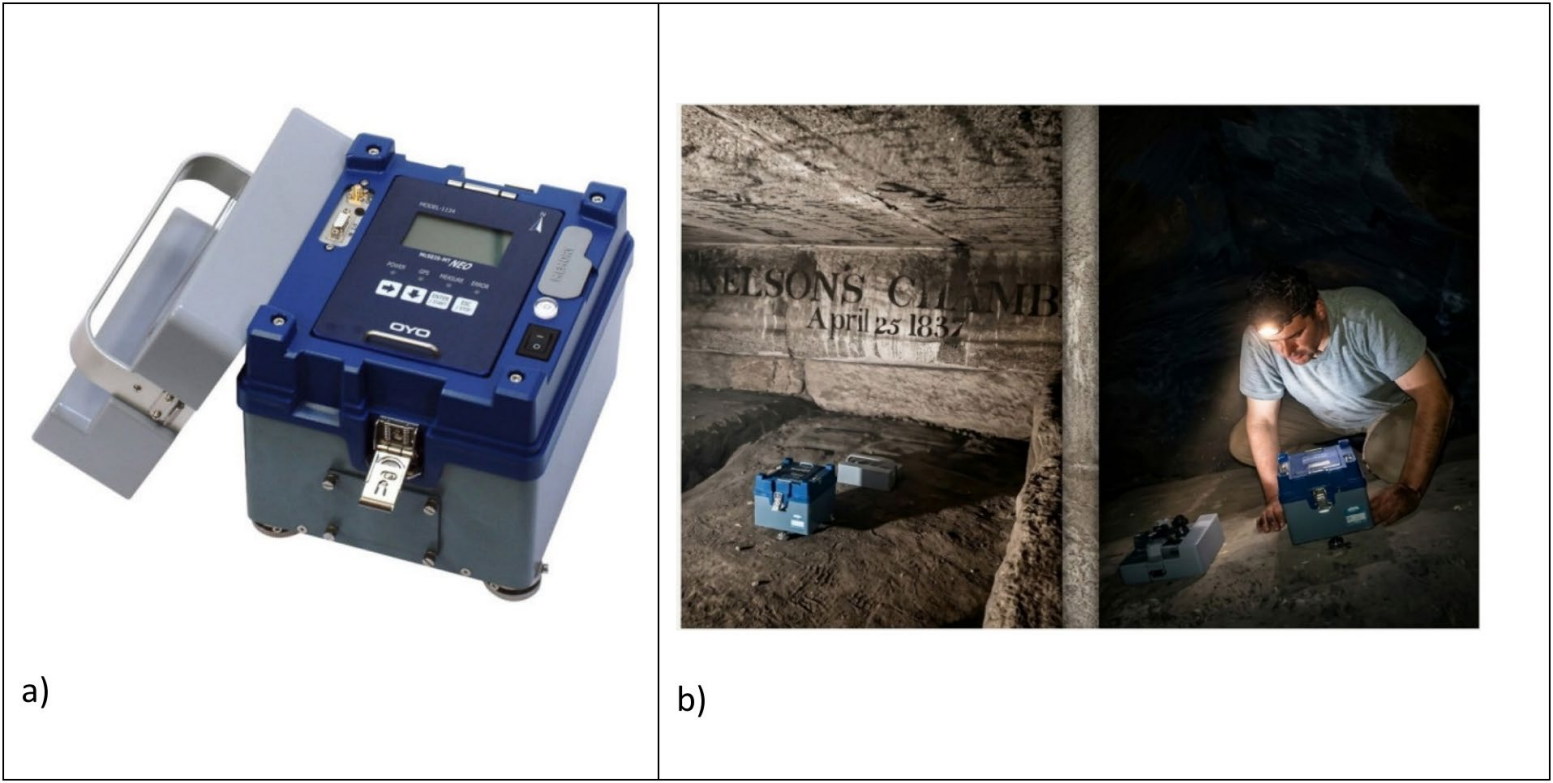
(**a**) McSEIS-MT NEO portable data acquisition accelerometer (adapted from OYO Corporation manual). (**b**) Field measurements in stress-relieving chambers located above the King’s Chamber.

This study analyses ambient noise measurements recorded inside the Great Pyramid of Khufu to characterise its dynamic and seismic behaviour. The main purpose of this study is to find out the fundamental frequencies for several monitoring positions inside the pyramid and to investigate whether the pyramid has a fundamental frequency similar to that of the surrounding ground as a way of recognising probable resonance phenomena. We used in our measurements the McSEIS-MT NEO portable data acquisition system developed by OYO Corporation in this survey (Fig. 3). This compact instrument integrates a high-sensitivity Servo-Accelerometer (3 axes, integrated in the main unit), with a global positioning system (GPS), and an internal battery within a single unit. The device operates over a frequency band of 0.1–200 Hz, with a sensitivity of 2.0 V/g and a dynamic range of 120 dB and resolution 1 μG^21,22^.

## Methods and techniques of measurements

Ambient noise measurements were carried out during this study in three field camps to recognise the dynamic response within Khufu’s Pyramid, particularly seismic action. Through this research, the H/V Spectral Ratio technique was used. This technique was widely used for extracting the fundamental frequency of a certain structure from noise measurements. The notable merit of the seismic ambient noise survey is that it is quite quick for inspection of complex structures, and its implementation is non-destructive. This technique is often utilised in the historical structures to refrain from the destructive survey, which may cause damage (e.g.^12^). The more conventional way to measure fundamental frequencies is to calculate the horizontal-to-vertical component spectral ratio (HVSR) from recorded seismic noise at a given site^12^**.** The “Nakamura method” is a way of determining both the fundamental resonance frequency f_0_ and the amplification of ground motions caused by a sedimentary surface layer at a given site^23^**.** Both fundamental frequency and relative amplification factor are obtained by dividing the spectrum of the horizontal components by the vertical component spectrum of ambient noise or earthquakes. The first peak of amplitude is a measure of (f_0_), which is directly linked to the disparity in the velocity of the shear-wave between the top sedimentary soft layer and the harder layer that underlies it. Depending on the research works of Lachet^24^ and Maklad et al.^25^**,** the amplification factor obtained from the maximum amplitude of the HVSR of microtremor measurements is not usable, while the peak frequency is applicable. The efficiency of the amplification factor is still under discussion^26^.

It was believed that the vertical component of the ambient noise preserves the characteristics of the source in the surface sediments that are comparatively influenced by the Rayleigh wave and can therefore be used to eliminate both the source and the Rayleigh wave impacts from the horizontal components. It is well noted that spectral features and seismic noise polarisation manifest a clear association with the geological site characteristics^27^. The spectral H/V microtremor ratio at frequency w,${\left(\mathrm{H}/\mathrm{V}\right)}_{\mathrm{m}}\left(\omega \right)$ is calculated using the equation of Arai and Tokimatsu^28^**.**

1 $${\left(\mathrm{H}/\mathrm{V}\right)}_{\mathrm{m}}\left(\omega \right)=\sqrt{\frac{{P}_{NS}(\omega )+{P}_{EW}(\omega )}{{P}_{UD}\left(\omega \right)}}$$

where *P*_UD_ (*x*) is the vertical component’s power spectrum and *P*_NS_(*x*) and *P*_EW_(*x*) are those of both horizontal components. Capon’s direct segment method^29^ is used to measure the Fourier power spectrum.

In this study, ambient vibration data were recorded using the McSEIS-MT NEO portable data acquisition system. The recording time is 15 min selected for each measurement point to ensure a sufficient signal-to-noise ratio for a reliable test of HVSR curves, following the SESAME European project, guidelines^30^. The analysis of HVSR was carried out using the Fortran program developed by Professor Takuni Hayashida, IISEE, Tsukuba, Japan. The Data analysis of HVSR technique includes the following steps; (1) separating data into blocks of time where each block includes 2048 points of data with a 100 samples per second sampling rate (window length 20.48 s); (2) implementing the Fast Fourier transformation to the three components noise waveform data to convert them into a frequency domain; (3) smoothing the three components noise spectra by employing the Parzan window filter of 0.25 Hz as a fixed constant for the selected 20.48 s window, ensuring that the spectrum is smoothed without losing important frequency details Yokoi^31^, (4) Estimating of the power spectrum for three-component noise records; (5) Calculation of the HVSR as the square root of the ratio of the two combined horizontal components to the vertical component power spectra^28^**,** (6) Monitoring changes in the HVSR with frequency. (7) The predominant frequency is defined as the frequency at which the first peak of the spectrum exists.

Ambient noise measurements allow us to calculate the Seismic Vulnerability Index (Kg), which shows how soil responds dynamically^32^. In this study, we computed Kg only for the surrounding soil at each measurement point outside the Pyramid. While Kg can relate to building damage risk, it’s not a direct safety measure for monuments^33^, Elbsheshi et al.^34,35^. Here, Kg simply indicates how easily the ground deforms at those locations.

Kg is useful for detecting the weak points of the ground. Kg value is estimated using the following equation:

2 $${\text{Kg }}\left( {\mathrm{e}} \right) \, = ({\mathrm{A}}_{{\mathrm{g}}}^{{2}} /{\mathrm{F}}_{0} )$$

where ‘Ag’ is the amplification factor and ‘F_0_’ is the fundamental frequency. The sites with Kg values greater than 20 are the most vulnerable^36^.

**Fig. 4:**
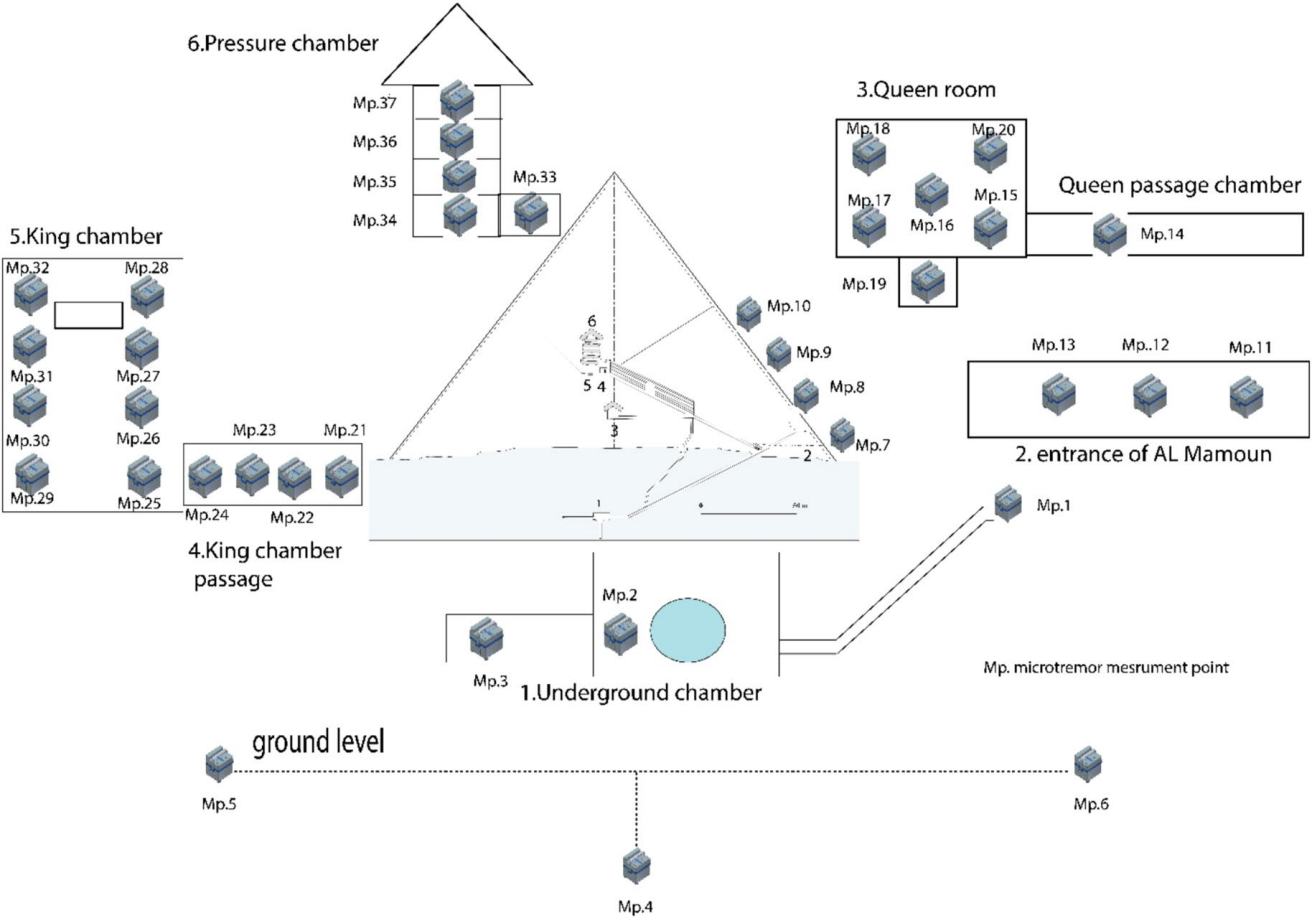
Sketch showing the noise measurement points inside the Khufu Pyramid.

Ambient vibrations were recorded at several sites in different locations inside Khufu’s Pyramid. The ambient noise measurements were conducted only inside the accessible areas at different levels. However, the research team encountered difficulties accessing the pressure chambers due to the narrow passage leading to them. All measurements were carried out when there were no human activities or tourist visits inside the pyramid. Figure 4 demonstrates the graphical presentation of the locations of the measurements inside the Khufu Pyramid. The ambient noise measurements have been conducted at 37 sites as follow; six measurements inside the Queen Chamber and another single measurements at its entrance passage, four measurements along the entrance passage of the King Chamber, eight measurements inside the King Chamber, three measurement at the soil close to the pyramid, single measurement at the starting point of Caliph al Ma’mun’s entrance, two measurement inside Caliph al Ma’mun’s passage, a single measurement at the entrance of the descending passage to the Subterranean chamber, a single measurement at Subterranean chamber, another single measurement inside the horizontal passage connected to the Subterranean Chamber, five measurements inside the pressure-relieving chambers and its entrance and four measurements on the outer stones of the pyramid with different heights.

## Results and measurements analysis

**Fig. 5:**
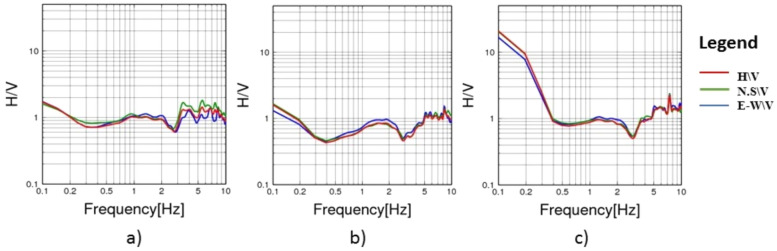
HVSR measurements at Subterranean Chamber under the Khufu Pyramid and the passage connected to it, (**a**) point (Mp. 1) with fundamental frequency 1.4 Hz at the entrance of descending passage, (**b**) point (Mp. 2) with fundamental frequency 1.3 Hz inside the chamber, (**c**) point (Mp. 3) with fundamental frequency 1.3 Hz inside the horizontal passage connected to it.

**Fig. 6:**
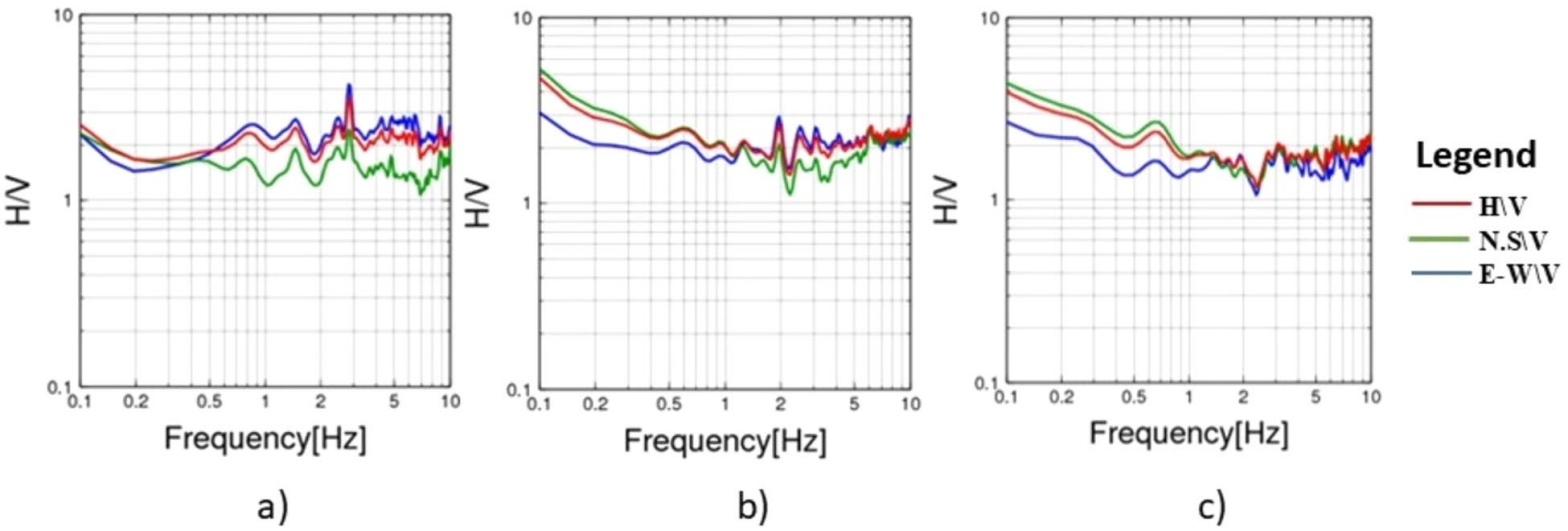
HVSR measurements at the ground in front of the pyramid, (**a**) point (Mp. 4) with fundamental frequency 0.6 Hz, (**b**) point (Mp. 5) with fundamental frequency 0.6 Hz, (**c**) point (Mp. 6) with fundamental frequency 0.63 Hz.

**Fig. 7:**
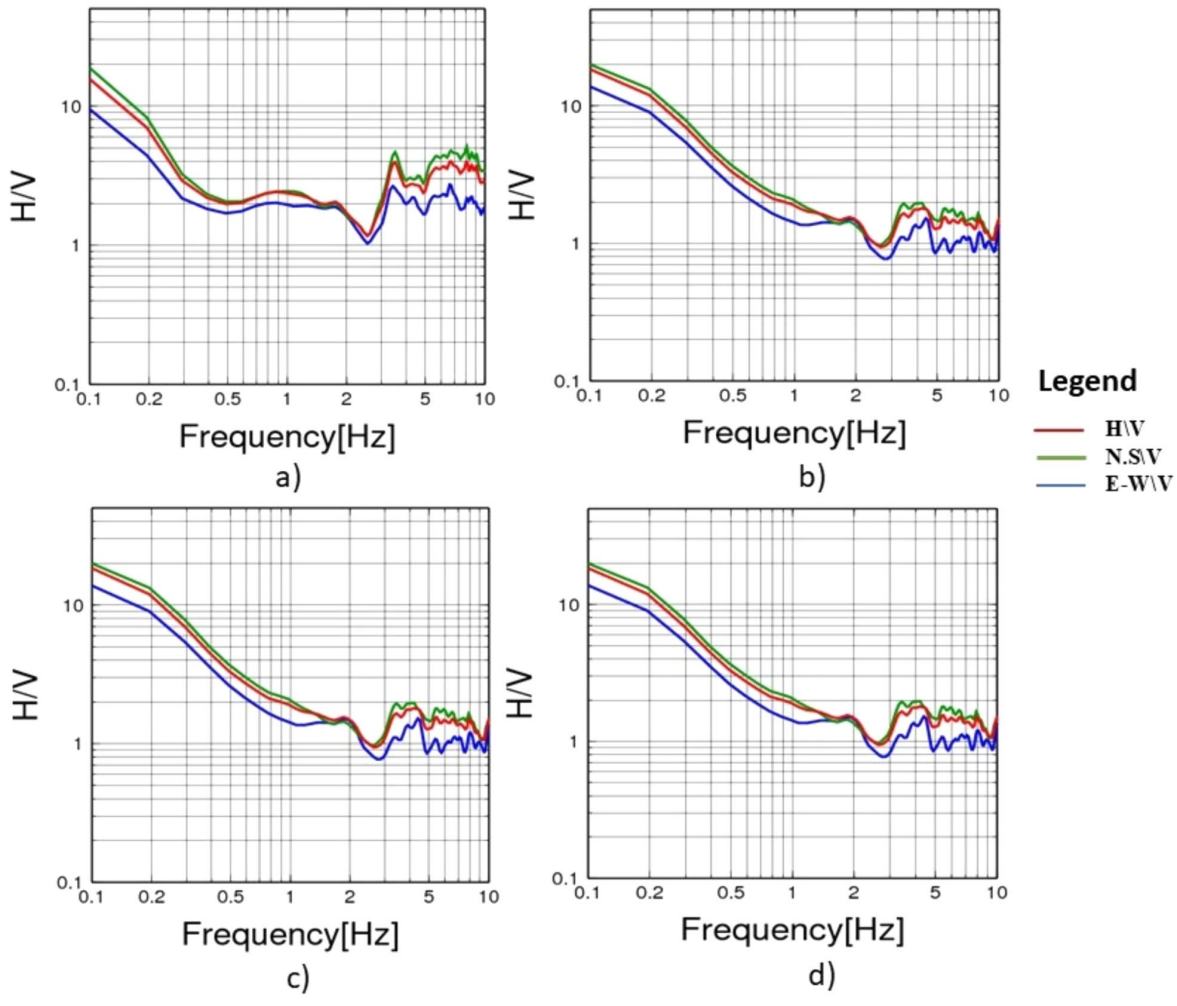
HSVR measurements on the outer stones of the pyramid, (**a**) point (Mp. 7) with fundamental frequency 0.8 Hz at the front of Caliph al Ma’mun’s entrance, (**b**) point (Mp. 8) with fundamental frequency 2 Hz, (**c**) point (Mp. 9) with fundamental frequency at 2 Hz, (**d**) point (Mp. 10) with fundamental frequency at 2 Hz.

**Fig. 8:**
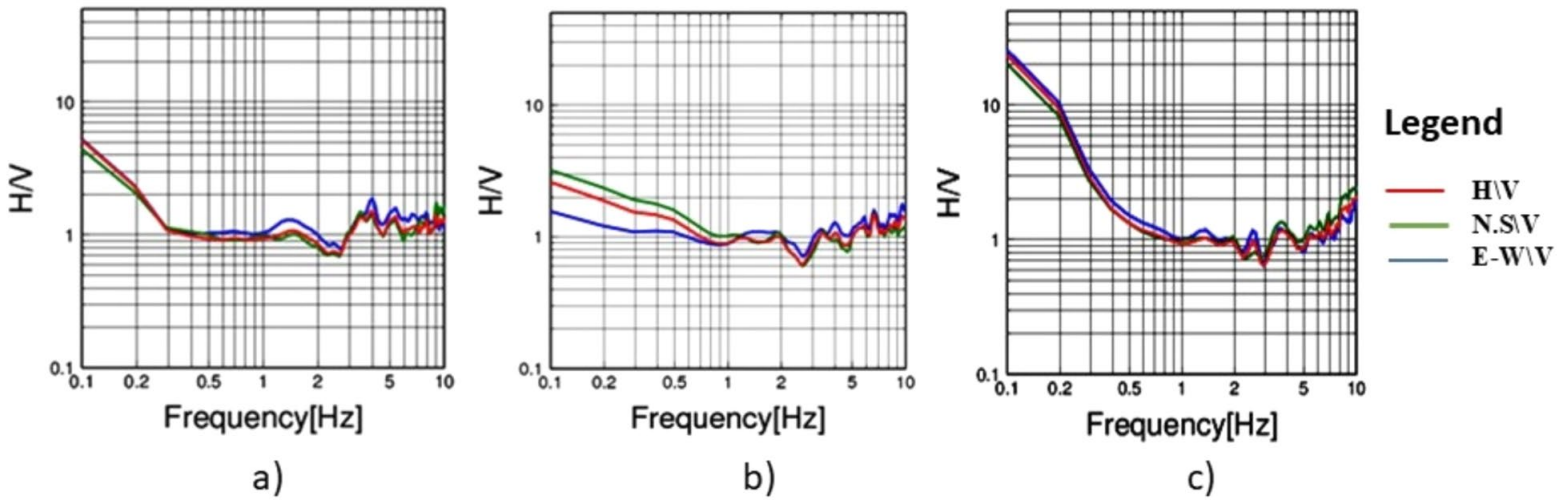
HSVR measurements inside Caliph al Ma’mun’s passage and its entrance, (**a**) point Mp. 11) with fundamental frequency at around 1.4 Hz at the entrance of Caliph al Ma’mun’s passage, (**b**) point (Mp. 12) with fundamental frequency 1.3 Hz inside Caliph al Ma’mun’s passage, (**c**) point (Mp. 13) with fundamental frequency 1.3 Hz inside Caliph al Ma’mun’s passage.

**Fig. 9:**
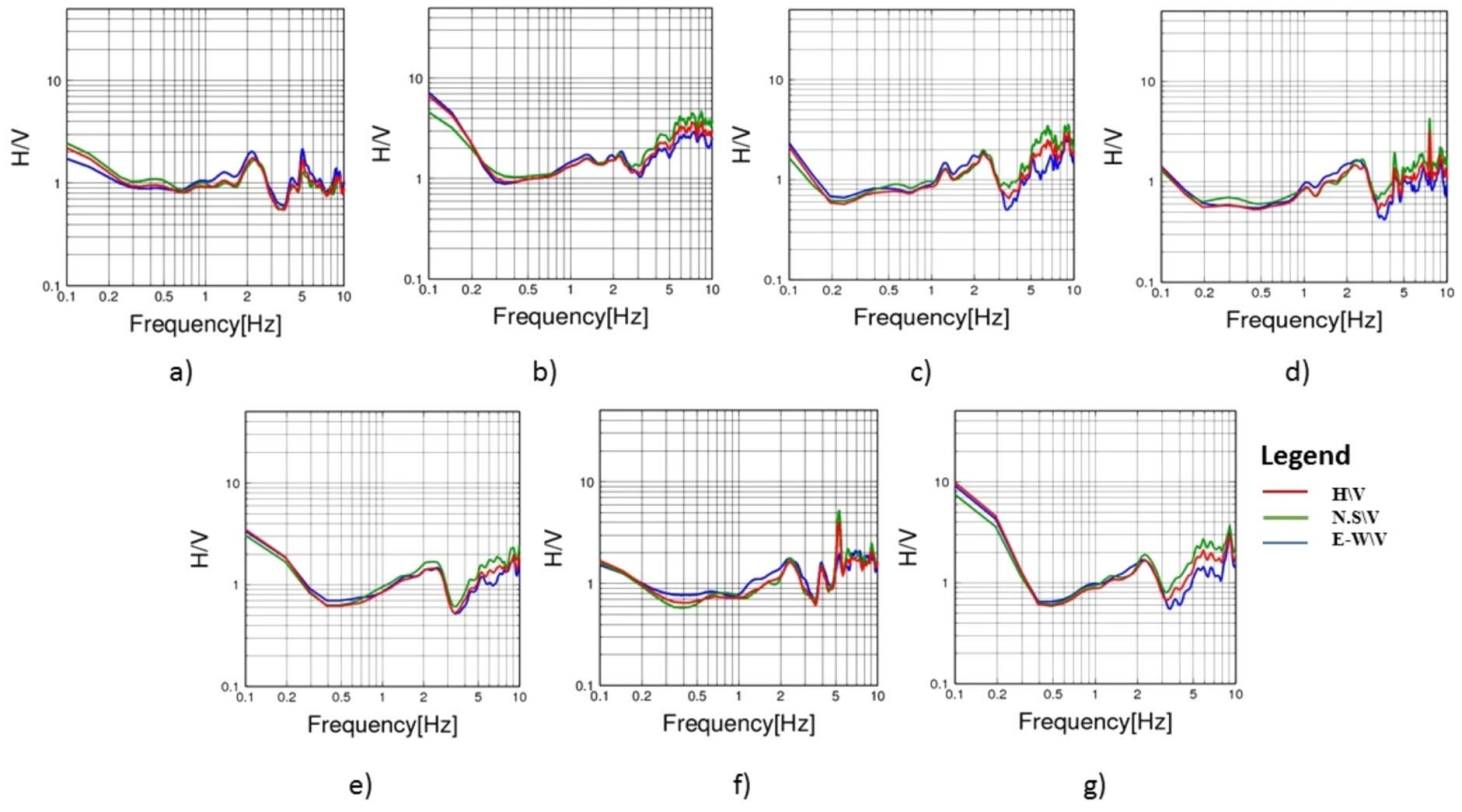
HSVR measurements at Queen chamber and its entrance a) point (Mp.14) with fundamental frequency 2.1 Hz at front of Queen chamber, (**b**) point (Mp.15) with fundamental frequency 1.5 Hz at the entrance passage of the Queen chamber, (**c**) point (Mp.16) with fundamental frequency 2.3 Hz inside the chamber, (**d**) point (Mp.17) with fundamental frequency 2.3 Hz inside the chamber, (**e**) point (Mp.18) with fundamental frequency 2.3 Hz inside the chamber, (**f**) point (Mp.19) with fundamental frequency 2.3 Hz at inside the chamber, (**g**) point (Mp.20) with fundamental frequency 2.3 Hz inside the chamber.

**Fig. 10:**
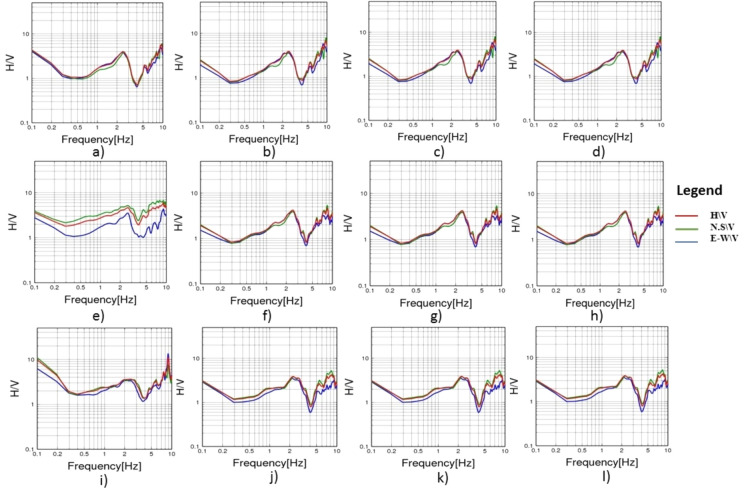
HVSR at different locations in King Khufu chamber and its entrance, (**a**) point (Mp.21) with fundamental frequency 2.4 Hz at the King Chamber passage, (**b**) point (Mp.22) with fundamental frequency 2.6 Hz at the King Chamber passage, (**c**) point (Mp.23) with fundamental frequency 2.6 Hz at the King Chamber passage, (**d**) point (Mp.24) with fundamental frequency 2.6 Hz at the King Chamber passage, (**e**) point (Mp.25) with fundamental frequency 2.6 Hz inside the King Chamber, (**f**) point (Mp.26) with fundamental frequency 2.4 Hz inside the King Chamber, (**g**) point (Mp.27) with fundamental frequency 2.4 Hz inside the King Chamber, (**h**) point (Mp.28) with fundamental frequency 2.4 Hz inside the King Chamber, (**i**) point (Mp.29) with fundamental frequency 2.3 Hz inside the King Chamber, (**j**) point (Mp.30) with fundamental frequency at 2.3 Hz inside the King Chamber, (**k**) point (Mp.31) with fundamental frequency 2.3 Hz inside the King Chamber, (**l**) point (Mp.32) with fundamental frequency at 2.3 Hz inside the King Chamber.

**Fig. 11:**
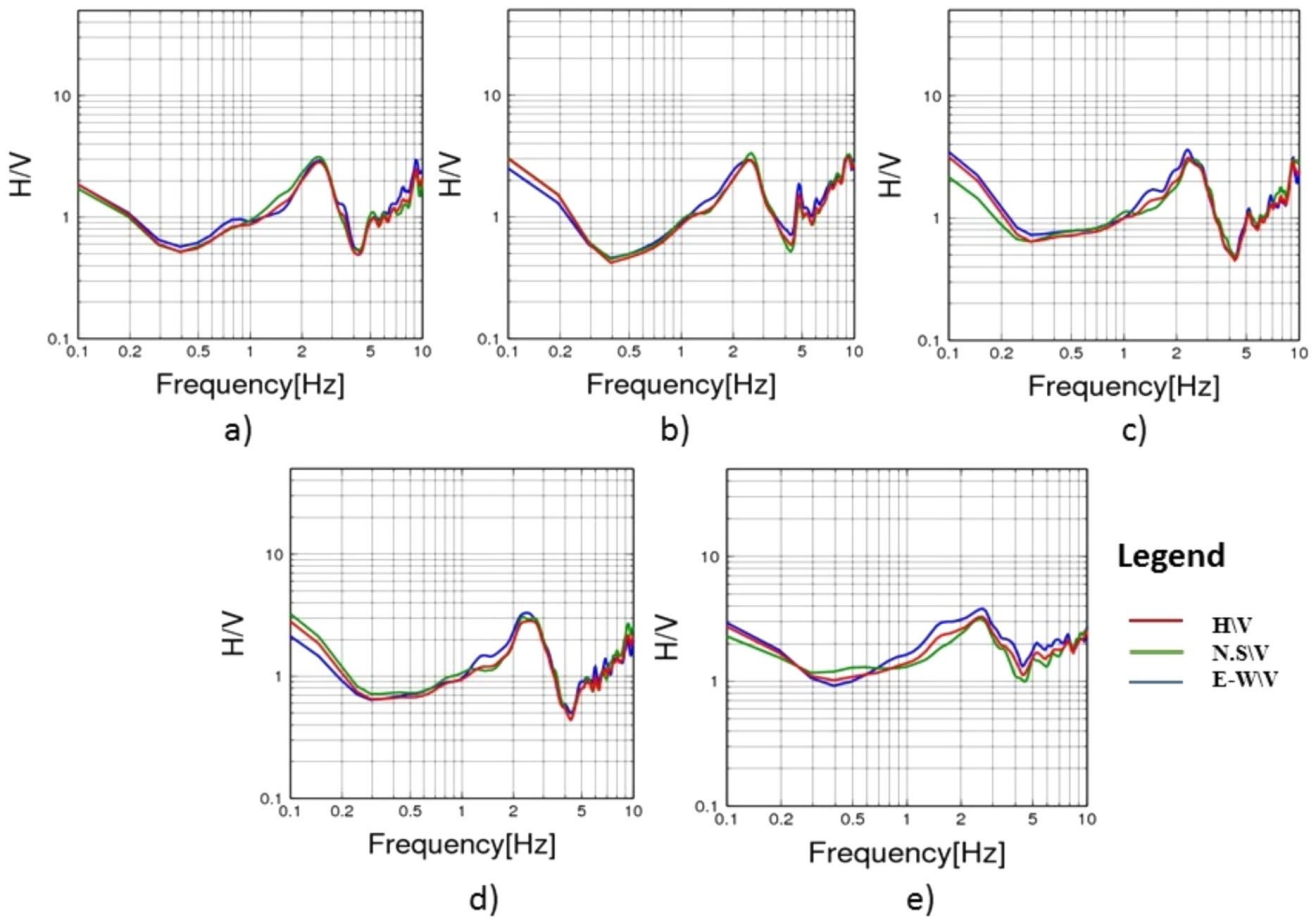
HVSR at the four pressure-relieving chambers, (**a**) point (Mp. 33) with fundamental frequency 2.4 Hz at the entrance tunnel, (**b**) point (Mp. 34) with fundamental frequency 2.4 Hz at the first pressure relieving chamber, (**c**) point (Mp. 35) with fundamental frequency 2.6 Hz second pressure relieving chamber, (**d**) point (Mp. 36) with fundamental frequency 2.4 Hz at the third pressure relieving chamber, (**e**) point (Mp. 37) with fundamental frequency 2.6 Hz at the fourth pressure relieving chamber.

To evaluate the dynamic seismic behaviour within the different elements of Khufu’s Pyramid, H/V results, which include the fundamental frequency (F_0_) and amplification (A_0_), have been summarised in Table 1. The estimated fundamental frequency (F_0_) is around 1.4 Hz at the entrance of the descending passage towards the Subterranean Chamber (Fig. 5a), while at the Subterranean Chamber and the horizontal passage connected to it, there is a slight decrease in the fundamental frequency (F_0_) to 1.3 (Fig. 5b, c). At the ground level in the front of the pyramid, the fundamental frequency peaks (F_0_) observed at three sites had a 0.6 value (Fig. 6a, b and c). Three of the four examined sites on the pyramid’s outside stones have a fundamental frequency value of around 2 Hz (Fig. 7b, c and d). The fourth measuring location, which lies in front of Caliph al Ma’mun, shows a lower fundamental frequency of about 0.8 Hz (Fig. 6a). The predominant frequency values of three measurement points along the passage of Caliph al-Ma’mun range between 1.3 and 1.4 Hz (Fig. 8a, b, and c). Among the seven measurement points inside the Queen chamber and its entrance passage, six points indicate fundamental frequencies (F_0_) ranging from 2.1 to 2.3. Measurements at the Queen chamber’s entrance manifest two peaks with two fundamental frequencies. The lower fundamental frequency is around 1.5 Hz (Fig. 9a, b, c, d, e, f and g) and Table 1. Another eight measurements inside the King chamber have been recorded along the two sides of the chamber in addition to four measurements along the King entrance passage. The fundamental frequency values of these measurements (F_0_) vary between 2.3 and 2.6 (Fig. 10a, b, c, d, e, f, g, h, I, j, k and l). Measurements conducted inside the four pressure-relieving chambers, which are located above the roof of the King chamber and its entrance passage, obtained equal fundamental frequency (F_0_ = 2.4) (Fig. 11a, b, c, d and e).

**Table 1:** The fundamental frequency and amplification factor inside the different structural elements of the pyramid.

No	Measurement point	Location inside the pyramid Khufu	Fundamental frequency (Hz)	Amp
1	Mp. 1	The entrance of the descending passage to the Substratum chamber	1.4	1.0
2	Mp. 2	Inside the Substratum chamber (under the pyramid)	1.3	1.0
3	Mp. 3	The small passage connected to the Substratum chamber	1.3	1.0
4	Mp. 4	At the ground in front of the pyramid	0.6	2.7
5	Mp. 5	At the ground in front of the pyramid	0.6	2.7
6	Mp. 6	At the ground in front of the pyramid	0.63	2.7
7	Mp. 7	Outer stones of the Pyramid in front of Caliph al-Ma’mun’s passage	0.8	2.4
8	Mp. 8	Outer Stones of the Pyramid	2.0	1.4
9	Mp. 9	Outer Stones of the Pyramid	2.0	1.4
10	Mp. 10	Outer Stones of the Pyramid	2.0	1.4
11	Mp. 11	Starting point of Caliph al-Ma’mun’s passage	1.4	1.3
12	Mp. 12	Inside Caliph al-Ma’mun’s passage	1.4	1.1
13	Mp. 13	Inside Caliph al-Ma’mun’s passage	1.3	1.2
14	Mp. 14	Queen chamber entrance passage (15 m from the starting point)	1.5	1.2
15	Mp. 15	Inside the Queen’s chamber	2.1	1.8
16	Mp. 16	Inside the Queen’s chamber	2.3	2
17	Mp. 17	Inside the Queen’s chamber	2.3	2
18	Mp. 18	Inside the Queen’s chamber	2.3	1.8
19	Mp. 19	Inside the Queen’s chamber	2.3	1.8
20	Mp. 20	Inside the Queen’s chamber	2.3	1.8
21	Mp. 21	In the entrance passage of the King’s chamber	2.4	4
22	Mp. 22	In the entrance passage of the King’s chamber	2.6	4
23	Mp. 23	In the entrance passage of the King’s chamber	2.6	4
24	Mp. 24	In the entrance passage of the King’s chamber	2.6	4
25	Mp. 25	King chamber	2.6	4
26	Mp. 26	King chamber	2.4	4
27	Mp. 27	King chamber	2.4	4
28	Mp. 28	King chamber	2.4	4
29	Mp. 29	King chamber	2.3	4
30	Mp. 30	King chamber	2.3	4
31	Mp. 31	King chamber	2.3	4
32	Mp. 32	King chamber	2.3	4
33	Mp. 33	Pressure room entrance tunnel	2.4	3
34	Mp. 34	Pressure room, first floor	2.4	3
35	Mp. 35	Pressure room, second floor	2.4	3
36	Mp. 36	Pressure room, third floor	2.4	3
37	Mp. 37	Pressure room, fourth floor	2.6	3

The estimated relative amplification factor for the ground level of the pyramid is equal to one. This value increases with height, with a maximum value of four (Table 1). The only exception is the pressure-relieving chambers, which deviate from this behaviour. Although these chambers represent the pyramid’s maximum studied height, they yield a lower value of about three. The seismic vulnerability index has been calculated for the measurements conducted at the ground in front of the Khufu pyramid. The obtained value of Kg is 8.2.

## Discussion and conclusions

The Horizontal-to-Vertical Spectral Ratio (HVSR) methodology is a non-destructive geophysical tool used in soil characterisation, structural analysis, seismic microzonation, and structural heritage. Several methodological, environmental, and physical constraints may impact the reliability and interpretation of its findings. Because of these limitations, HVSR is not an absolute measure of motion amplification within structures, but rather a relative spectral ratio. It represents the frequency-dependent ratio between horizontal and vertical components, not the true building transfer function^37^. Instead, it serves primarily as an indicator of modal response variations^38^. Further constraints may encompass the complex geology^39^, industrial noise necessitating meticulous precautions during field measurements^40^, difficulties in detecting low frequencies within deep sediments^25^, and alterations to ground motion induced by buildings. Gallipoli et al.^41^ show that measurements within structures often yield distorted HVSR curves, whereas soil-structure interaction can produce mixed spectral peaks that hinder the differentiation between soil and building resonances.

In this study, we investigated ground-structure interaction effects on the Khufu Pyramid by analysing its fundamental frequency and relative amplification factor. Thirty-seven ambient noise measurements have been recorded at elevations up to ~ 61 m across the various elements of the Khufu pyramid and the surrounding soil. The majority of ambient noise measurements (76%) from different elements within the pyramids exhibit a fundamental frequency between 2.0 and 2.6, with an average of ~ 2.3 in the whole Pyramid structure. This means that the dynamic properties of the investigated structure are homogeneous. The homogeneous distribution of stress across the pyramid’s structural elements is related to proper torsional resistance and stiffness, which reduce torsional motion^8^. The reduction of torsion results from the existence of both the centre of mass and the resistance at the same location or close to the same point. In contrast, the average predominant frequency on the surrounding ground is about 0.60 Hz, reflecting Giza plateau geology. This frequency mismatch between the pyramid and soil aligns with the pyramid’s observed 4,600-year resilience against nearby earthquakes, during which no serious structural damage occurred. The presence of a second peak on ground surfaces likely stems from multiple alternating layers of varying lithology, rather than horizontal stratification in homogeneous layers^24,27,42–45^.

The underground subterranean chamber and the small passage connected to it manifest a fundamental frequency value of about 1.3, with a relative amplification factor equal to one. The lack of a distinct peak in the H/V curves at this frequency indicates that either you are directly on bedrock or the sedimentary layer above it is extremely thin. An amplification factor equal to one implies that there is no amplification, which usually correlates with the existence of the bedrock. In front of Al-Khalifa Al-Mamun passage, the fundamental frequency value on the pyramid’s exterior stones drops suddenly (0.8) when compared to other outside measurements on stones located at higher elevations (2.0). This small value is nearly consistent with the predominant frequency on the ground, illustrating how this location is linked to the ground underneath the pyramid. There is no correlation between the fundamental frequencies along the passage of Caliph al Ma’mun (1.3–1.4 Hz) and the other structural elements in the pyramids (2–2.6 Hz). This passageway, which was later excavated and was not included in the pyramid’s original design, may be the cause for this drop in the fundamental frequencies.

 It is noticed that the estimated values of the relative amplification factor vary from 1 to 4, switching from the ground level to the King chamber elevation. This means that the relative amplification in the different structural elements increases with increasing their heights. A reduction in the amplification factor exists at the pressure-relieving chambers, which are located above the King Chamber and represent the maximum investigated heights within the pyramid. This result is consistent with the idea that the design of these rooms contributes to diminishing the stresses on the King Chamber. Some measurement points give comparatively anomalous fundamental frequencies (Fo) compared to the dominant one in the majority of the structural elements inside the pyramid (Table 1).

The Seismic Vulnerability Index (Kg) in this study assesses soil vulnerability only, not the structural safety of the entire stone pyramid monument. Current evidence doesn’t support using soil-based Kg values to evaluate large stone structures, as this would require explicit methodology linking soil parameters to internal monument dynamics. Our soil Kg value of 8.2 (< 20) indicates a low site vulnerability index per standard physical classification, but doesn’t predict pyramid structural damage. The pyramid is distinguished by certain geometric aspects and features from an engineering point of view that make it one of the best designs resistant to earthquakes^8^. Among these features is the pyramid’s construction on hard limestone, which was a good example of constructing a tall building on hard rock to increase earthquake resistance. Having the largest mass percentage close to the ground and a decreasing percentage as we go up is another geometrical feature.

The observed frequency separation between soil (~ 0.6 Hz) and pyramid structure (~ 2.3 Hz) indicates naturally reduced resonance risk, which may contribute to the monument’s remarkable seismic endurance over millennia. Any suggestion of intentional seismic optimisation by ancient Egyptian architects remains purely speculative and cannot be substantiated by geophysical measurements alone. “HVSR captures only the dominant soil-structure resonance frequency and does not resolve higher-order local modes nor full modal characterisation for the Egyptian Khufu Pyramid. Which would require dedicated modal identification techniques such as ambient vibration testing or operational modal analysis (OMA). **Our future works** will include repeating of some measurements at key locations which gave little anomalies variations in fundamental frequencies (Fo), full soil-structure interaction (SSI) analysis with coupled numerical modelling, frequency content of earthquake input motion, damping ratio, and directional effects, plus operational modal analysis (OMA), or finite element modelling to validate mode shapes and quantify uncertainties. These findings will affirm the Khufu Pyramid as both an architectural marvel and a testament to ancient seismic engineering principles relevant to modern geoheritage conservation.

## Data Availability

All data and related results of this article are presented in the paper, and any additional materials can be requested from the authors.
